# Restoration of Severe Tooth Wear: Converting the Splint Jaw Position Into Definitive Restorations

**DOI:** 10.1111/jerd.13479

**Published:** 2025-04-30

**Authors:** Tuba Aini, Jan‐Frederik Güth, Kathrin Seidel, Steffani Görl, Tobias Graf

**Affiliations:** ^1^ Department of Prosthodontics, Center for Dentistry and Oral Medicine (Carolinum) Goethe University Frankfurt am Main Frankfurt am Main Germany

**Keywords:** bite splint, full mouth rehabilitation, temporomandibular dysfunction, tooth wear, vertical dimension of occlusion

## Abstract

**Objective:**

Loss of the vertical dimension of occlusion is observed in patients with severe tooth wear and often requires comprehensive prosthodontic treatment. A critical challenge is ensuring the precise transfer of the initially established jaw relationships to the definitive restorations. This study aimed to describe several approaches for simplifying the implementation of pretherapy into final restorations.

**Clinical Considerations:**

In the initial phase, structured treatment planning is essential to minimize potential sources of error. Prior to the placement of definitive dentures, a preliminary test phase using occlusal splints or long‐term provisional restorations is crucial for achieving optimal predictability of treatment outcomes. Noninvasive therapy with splints is generally employed as the first step following a diagnostic mock‐up. Subsequently, a test phase involving long‐term temporaries may be implemented to comprehensively evaluate functional, esthetic, and phonetic parameters. Consequently, the application of long‐term temporaries facilitates the accurate clinical transfer of the validated bite position to the definitive restorations.

**Conclusions:**

Multiple treatment approaches can be viewed as module components that can be combined to tailor therapy according to the complexity and needs of the patient. This strategy enables the most predictable outcome in terms of function and esthetics, while ensuring safe transfer of vertical and horizontal bite relationships and preserving tooth structure.

**Clinical Significance:**

Implementing the newly developed and tested occlusal relationship into definitive restorations requires thorough diagnostics, a structured and preferably noninvasive pretreatment approach, and a reliable method for transferring the tested bite relationship.

## 1 Introduction

**FIGURE 1:**
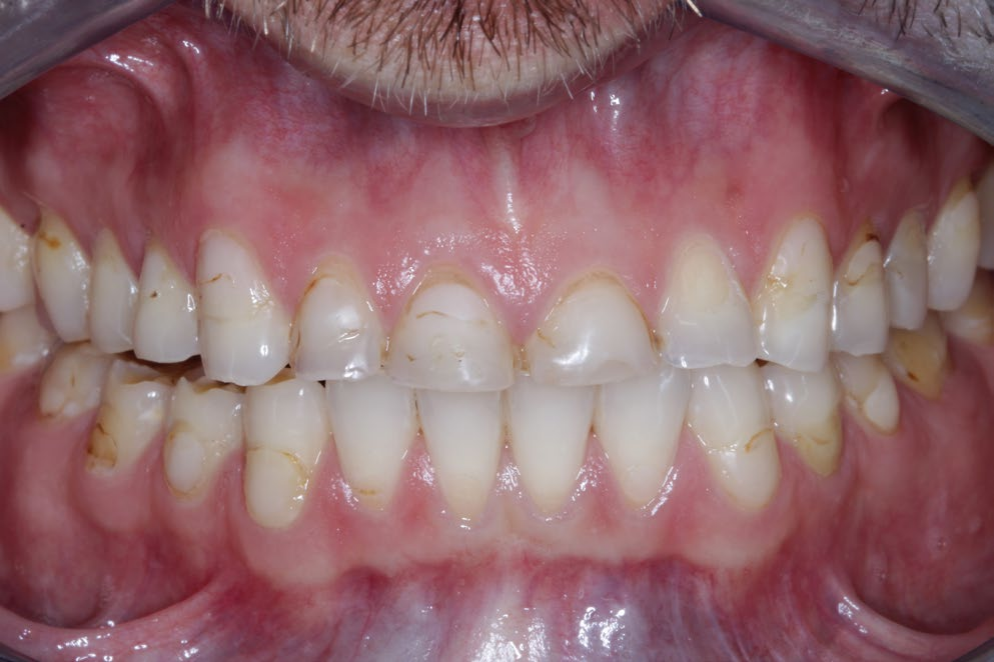
Initial clinical presentation of a patient with severe tooth wear.

Tooth wear is a common finding in dental practice and is often associated with the loss of the vertical dimension of occlusion (VDO, Figure 1). Its prevalence varies depending on factors such as age, presence of shortened dental arches, diagnostic criteria, and ethnic background (e.g., Arabic, Chinese, and European) [1, 2, 3, 4, 5].

When restorative or prosthodontic treatment is required, the initial step typically involves a trial phase using bite splints or long‐term temporaries (LTT). Detailed planning is essential to ensure predictable therapeutic outcomes [6, 7, 8, 9], including the analysis of temporomandibular joint (TMJ) function. Generally, patients undergo an extended pretreatment phase lasting several months before any invasive and irreversible restorative procedures are performed [7, 10, 11, 12, 13]. Therefore, various treatment approaches have been proposed to guide the transition from initial therapy to definitive restorations [14, 15, 16, 17, 18, 19].

First, a patient‐specific evaluation of function, esthetics, and phonetics is essential for the reliable prediction of the new therapeutic bite position. One of the ongoing challenges during treatment is the transfer of this newly defined mandibular position to fixed definitive restorations without compromising the established bite position or vertical dimension.

**FIGURE 2:**
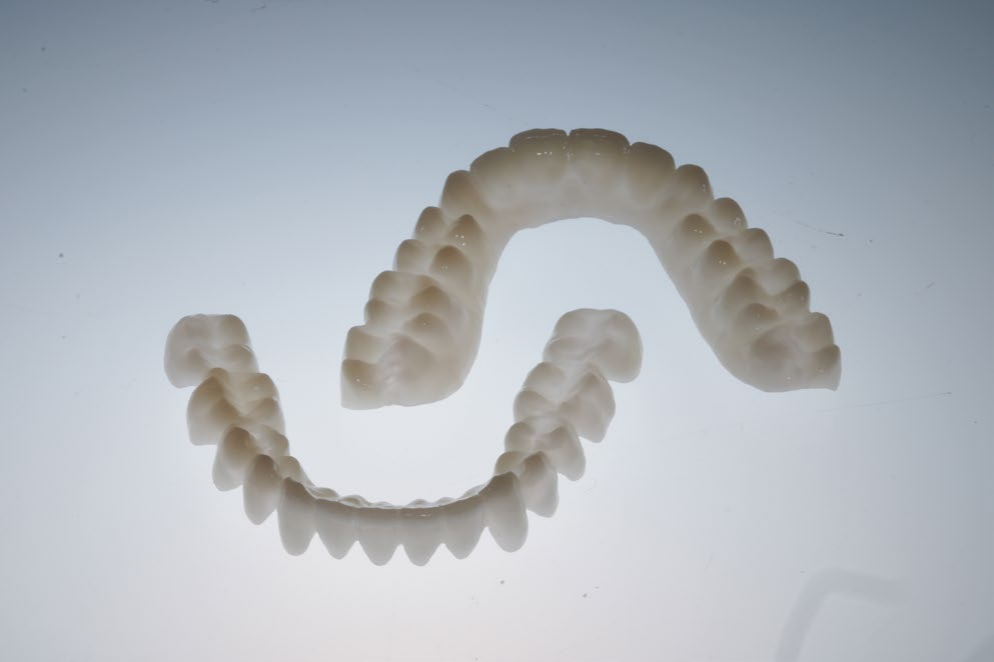
Polycarbonate splints.

**FIGURE 3:**
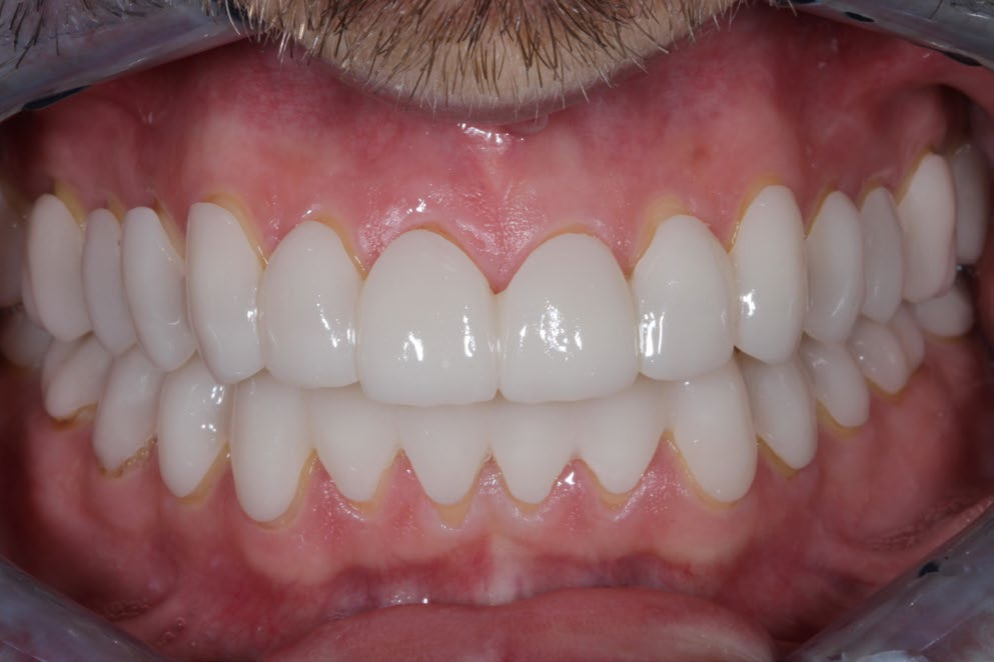
Polycarbonate splints in the same patient shown in Figures 1 and 2, with adjustment vertical dimension of occlusion.

**FIGURE 4:**
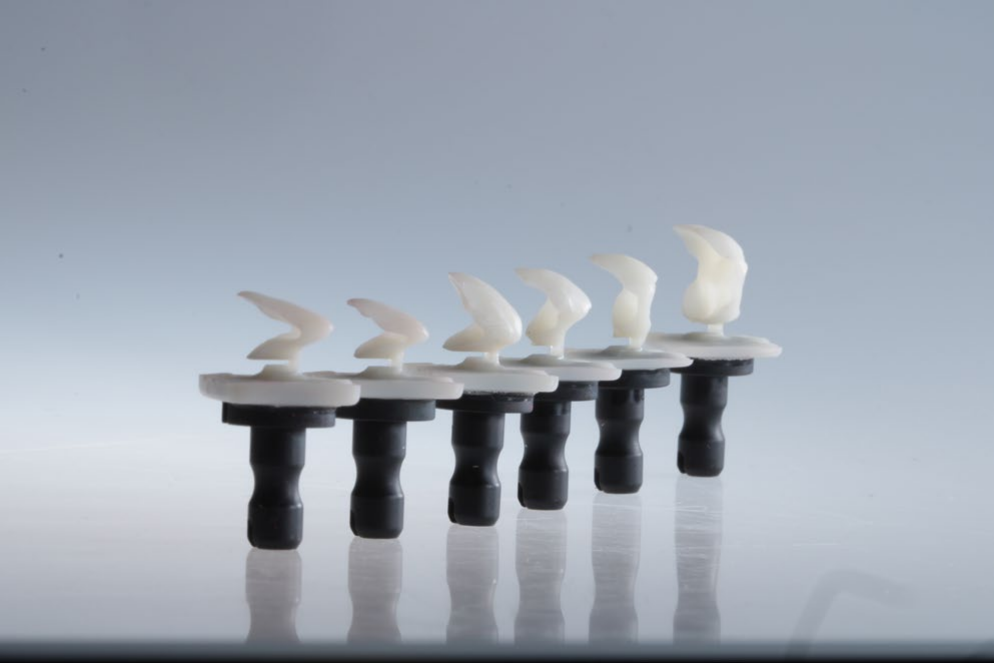
Non‐prep LTT restorations; material: Lava Ultimate in shade A1 (3M Espe, USA).

**FIGURE 5:**
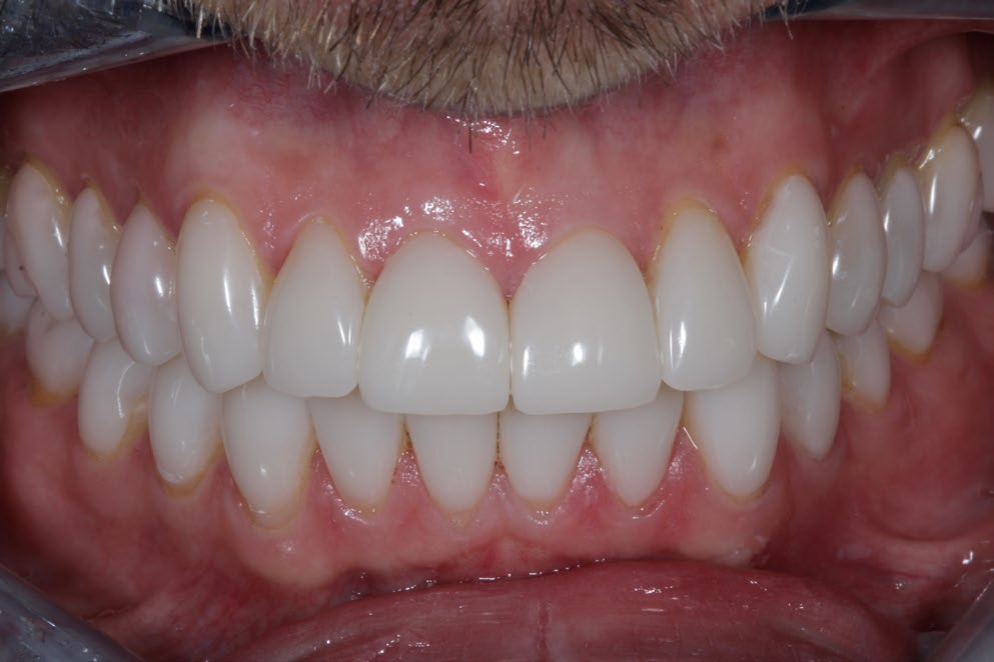
Noninvasive therapy using non‐prep LTTs as shown in Figure 4 (Lava Ultimate A1, 3M Espe).

**FIGURE 6:**
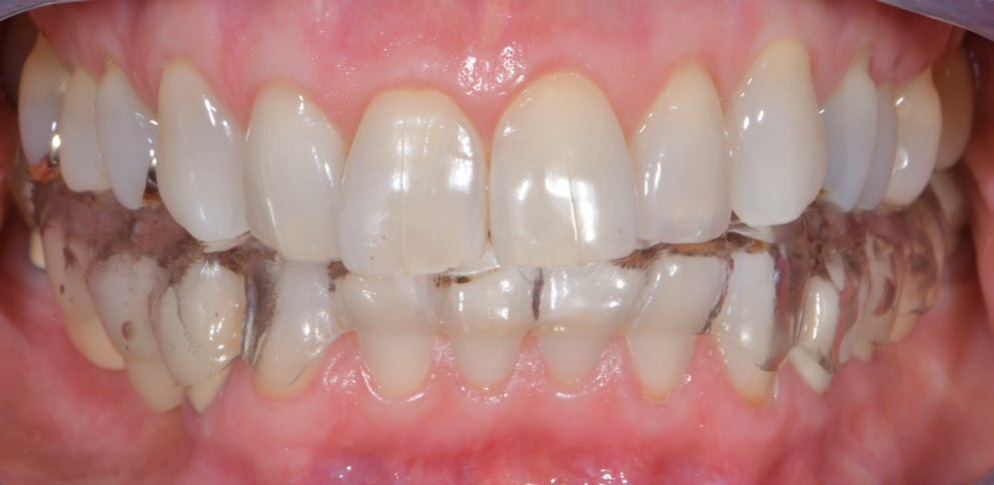
Monomaxillary PMMA splint (upper jaw) used as splint therapy at the newly determined vertical dimension of occlusion.

**FIGURE 7:**
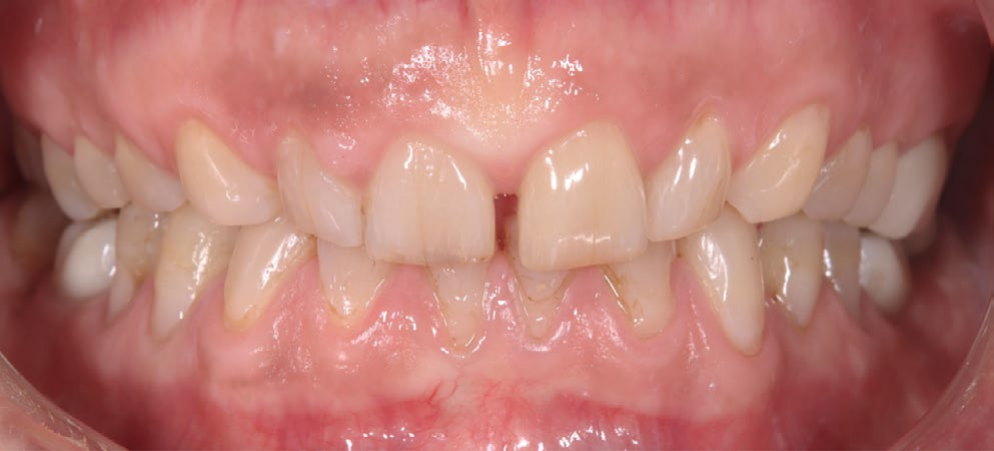
Patient before treatment with bimaxillary polycarbonate splints.

**FIGURE 8:**
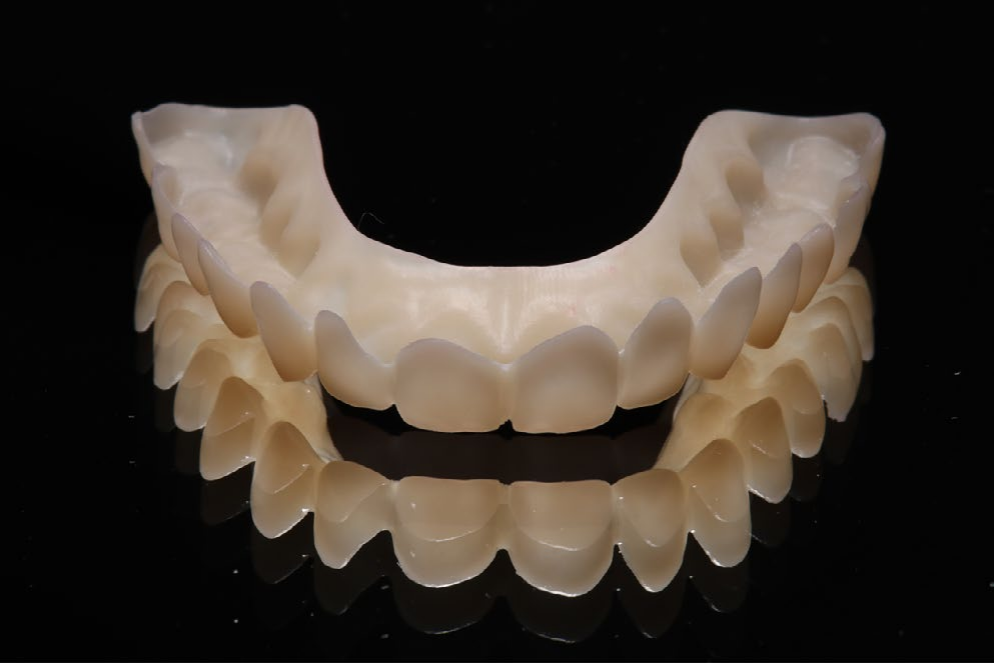
Polycarbonate splint (upper jaw) with palatal enforcement.

**FIGURE 9:**
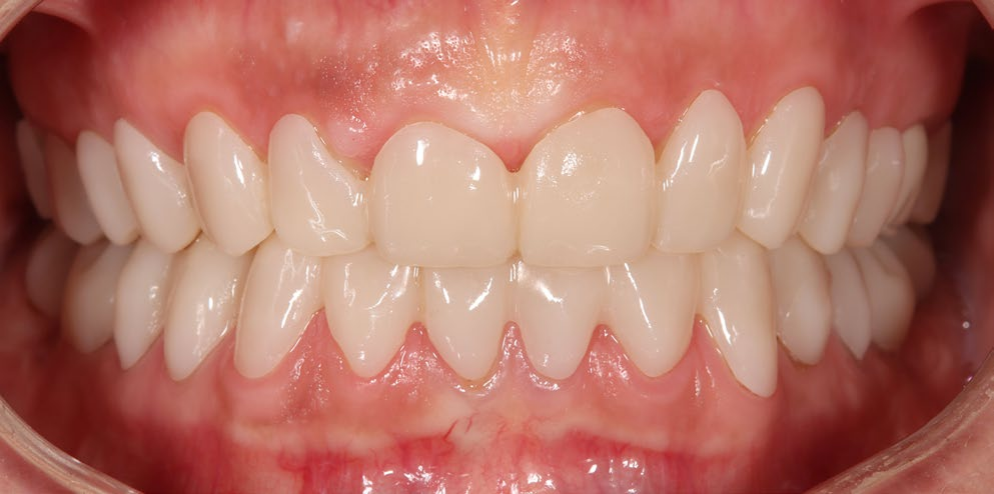
The same patient as in Figure 7 treated with bimaxillary polycarbonate splints.

To address this, new dental technologies and materials have been introduced, offering valuable support during the initial stages of treatment [20, 21]. Bite splint and/or long‐term temporary restorations that closely replicate the geometry of the planned final restorations contribute to a predictable and safe workflow (Figures 2, 3, 4, 5, 6, 7, 8, 9). Invasive procedures should be postponed until the final transposition into definitive restorations is ready to be performed. This study aimed to outline and discuss potential pathways for safely and predictably transferring an evaluated splint position into definitive restorations.

## 2 Concept: Diagnostics, Pretreatment, and Definitive Denture

### 2.1 Functional Findings: Parameters to Define the New Vertical Dimension of Occlusion

To date, no universally reliable parameters exist for precisely defining the VDO. Instead, the concept of a “comfort zone” has been introduced, representing an individual's physiological tolerance range. This zone provides functional and esthetic flexibility, allowing for adequate treatment planning and execution [22, 23].

The primary indications for changing the VDO are as follows:

- Providing space for restorations.
- Enhancing dentofacial esthetics.
- Restoring and improving occlusal relationships.
- Preserving further tooth substance loss.

Dentate patients typically exhibit lower adaptability and acceptance when establishing a new VDO compared with edentulous patients, owing to the need to reconstruct existing teeth and reestablish the occlusal plane [23]. To date, no specific quantifiable parameter or standardized guideline exists to determine the extent to which the VDO should be increased following the loss of tooth structure. Therefore, clinicians must establish a new VDO that balances facial and dental esthetics, functional requirements, and biomechanical considerations of the planned restorations, while adhering to the principle of minimally invasive therapy [11]. In clinical practice, the following parameters can aid in determining a new, physiologically appropriate bite height [8]:

- *Shimbashi‐Relation*: The Shimbashi Relation is assessed by measuring the distance from the gingival margin of the upper incisor to that of the lower incisor [24, 25, 26].
- *“Freeway space” (FWS)*: This space refers to the interocclusal distance between the upper and lower jaws when the mandible is at rest. It is determined by muscular activity and varies among individuals [27].
- *Phonation*: “S” or “F” sounds can serve as functional guides in evaluating the new VDO [27].
- *Lip and face profile*: This serves as a visual guide for assessing the inclination of the anterior teeth and a key esthetic parameter in determining the VDO [28].
- *Kollmann proportions*: These proportions denote the division of the face into three equal vertical parts—the forehead, nose, and jaw. A shortened lower third of the face indicates loss of VDO [28].

A permanent increase in the vertical dimension (following its loss) of up to 5 mm (measured in the incisor region) is a safe, predictable, and well‐tolerated procedure [8, 15]. The stomatognathic system adapts effectively to moderate changes in the VDO [10]. However, certain clinical scenarios necessitate the evaluation of both the new VDO and the horizontal bite position. In cases involving temporomandibular disorder (TMD), significant changes in bite height, or an uncertain bite, a preliminary, noninvasive pretreatment testing phase—such as the use of occlusal splints or long‐term provisional restorations—is recommended as part of an exemplary treatment approach.

The pretreatment phase was intended to ensure the absence of pain associated with craniomandibular disorders and to simulate the new bite position in terms of functional, esthetic, and phonetic parameters. Through this extended pretreatment phase, potential complications could be identified and addressed early by adjusting the bite position or modifying the contours of the restorations, thereby minimizing both clinical and financial burdens. The most appropriate therapeutic approach and modality should be selected based on the primary treatment objective.

### 2.2 Temporary Phase for Raising the VDO With Splints

During the provisional phase, the new bite position was evaluated using an occlusal splint. Typically, two different splint systems may be employed in the initial treatment: the “classical” transparent mono‐maxillary splint and the “advanced” bimaxillary splint, which mimics natural tooth color and form. Both approaches offer two significant advantages: complete reversibility and noninvasiveness.

#### 2.2.1 “Classic”: Monomaxillary Bite Splint

This type of splint is typically manufactured from polymethyl methacrylate (PMMA), a hard, rigid, and usually transparent material. It is placed monomaxillarily, either in the maxilla or mandible, and designed with anterior and canine guidance (Figure 6). The splint may be produced through milling, three‐dimensional (3D) printing, or manual techniques. Regardless of the fabrication process, PMMA exhibits a modulus of elasticity of 1.8 GPa and an average flexural strength of 55 MPa, according to the manufacturer's specifications (Table 1), with a minimum layer thickness of 1.0 mm. These splints are constructed with an adjusted occlusal surface to facilitate the establishment of a new VDO and/or a new horizontal jaw relationship. From a functional perspective, the contact zone between the opposing dentition and the PMMA splint differs in level from that of the final restorations or the previous wax‐up/mock‐up. As a result, esthetic and phonetic outcomes may be limited, potentially leading to reduced patient compliance [23, 29].

**TABLE 1:** Comparison of the PMMA splint and polycarbonate splint.

	The classic (PMMA splint)	The advanced (polycarbonate splint)
Features	Transparent PMMA	Tooth shades Polycarbonate
Advantages(+)	Many years of experience Good influence on function Adjustment only possible in function	Design and color (esthetics) Limited influence on function, good influence on esthetics and phonetics Flexible ➔ insertion direction compensable Free from MMA
Disadvantages(−)	More solid design (esthetics, phonetics or pronunciation limited) Polymerization shrinkage Possible residual monomer content Compliance rather poor	Low retention ➔ Not recommended to wear while eating Two splints necessary for bimaxillary restoration In some cases of TMD, pretreatment with PMMA splint is necessary More cost‐intensive for patient BPA content
Material properties	E Modulus 1.8 GPa Flexural strength 55 MPa	E Modulus 2.4 GPa Flexural strength 100 MPa

The transfer of the tested splint position to the definitive restorations is considered a further challenge when this type of splint is used.

#### 2.2.2 “Advanced”: Bimaxillary Polycarbonate Splint

The polycarbonate splint, characterized by its tooth‐colored appearance, offers favorable material properties and increased flexibility. It possesses a modulus of elasticity of 2.4 GPa and an approximate flexural strength of 100 MPa (Table 1), with a minimum required thickness layer of approximately 0.5 mm. These splints can be anatomically designed as mono‐ or bimaxillary, depending on the restorative approach, and are typically defined through a wax‐up/mock‐up and milled from a polycarbonate blank. This type of splint enables the simulation of functional parameters—including the new VDO and jaw relationships—as well as phonetic and esthetic aspects. Patient preferences may be incorporated at this stage, contributing to increased acceptance. The tooth‐colored appearance is associated with higher patient compliance, with daily wear recommended for up to 24 h during the pretreatment phase. However, wearing this type of splint while eating is not recommended. Patients are generally able to speak with a splint without significant restrictions and may adapt to the new tooth morphology with their social environment (Figures 3 and 9). Additionally, palatal and lingual enforcement can be incorporated due to the removable nature of the splint (Figure 8).

### 2.3 From Splint to Restoration

Both types of splints should be worn for at least 3–6 months to evaluate the new VDO. Until the splint position is transferred into fixed restorations, the treatment remains minimally invasive, with an adjustable and largely reversible occlusion. The method of transferring the splint position to definitive restorations varies depending on the splint morphology. There are different methods for the transfer, and this may present specific challenges. The most complex and demanding approach involves the direct placement of definitive restorations following the splint pretreatment phase. Alternatively, an intermediate step involving the use of minimal or nonprep LTT can be employed. These temporaries can be efficiently fabricated from composites, PMMA, or other polymer‐based materials through milling or 3D printing (Figure 4) and subsequently luted onto the teeth. The design of non‐prep LTT is guided by the newly established occlusal height and the previously defined wax‐up or mock‐up. However, modifications can be made to accommodate individual patient preferences.

High‐precision impressions and the use of a facebow are required for the delivery of LTT. Sharp edges of the teeth should be restored, and any existing restorations of inadequate quality must be replaced. A primary challenge lies in the accurate transfer of the tested, optimal, and functionally safe vertical and horizontal bite positions to the definitive restorations. The specific approach to this transfer may vary depending on the splint type and the nature of planned restorations.

### 2.4 Various Paths: One Aim

#### 2.4.1 1A: Monomaxillary PMMA Splint: Definitive Restorations

**FIGURE 10:**
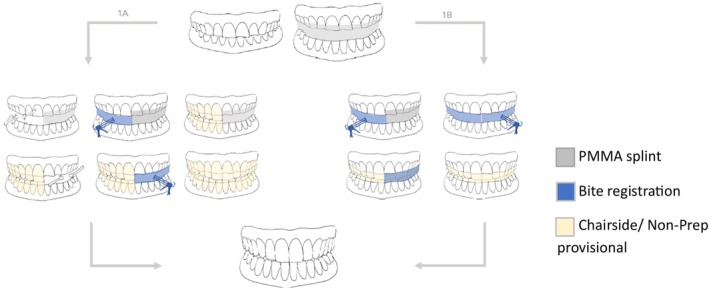
From PMMA splint to final restoration (left: Path 1A, right: Path 1B).

In this approach, the PMMA splint is segmentally shortened, allowing antagonistic preparation in the region where the splint has been removed, such as FDI 17/47 and FDI 16/46 (Figure 10). To assess interocclusal clearance and available restorative space, the shortened PMMA splint is inserted during treatment, and interocclusal registration is carried out over the completed antagonistic preparations. Following the completion of each preparation, the splint is shortened, and the corresponding registration is performed sequentially. The sequence and extent of stepwise reduction may be modified based on the clinician's experience, case complexity, and patient compliance. Once the teeth are prepared, chairside temporaries are fabricated at the newly established bite position and verified against the occlusal scheme of the splint. The division of treatment into multiple appointments is often necessary, as the shortened splint may not offer sufficient retention for secure intraoral placement. To support the fabrication of temporaries at this stage, a mockup representing the tested position can be advantageous. A bonded mock‐up, in particular, may serve as a practical solution, as it allows occlusal testing in relation to the splint position and can reduce the intensity of preparation sessions. Consequently, careful planning of preparation appointments—in terms of time allocation and equipment availability—is essential. Nevertheless, long sessions are exhausting for both patients and dentists. Therefore, the transfer from a monomaxillary splint to definitive restorations is considered a challenging process.

#### 2.4.2 1B: Monomaxillary PMMA Splint—Non‐prep LTT—Definitive Restorations

The application of LTTs as an intermediate step can alleviate many of the procedural challenges and enhance patient comfort throughout the treatment process (Figure 10). Nevertheless, an antagonistic approach is still required to verify the occlusal relationships using the existing splint. For this purpose, the splint is also shortened, and the non‐prep LTTs—fabricated at the new VDO—are antagonistically luted (e.g., teeth 11–17 and 41–47). Subsequently, the inserted LTTs stabilize the VDO, while the opposing quadrants are restored with the corresponding LTTs in the subsequent process. Following full placement, a test and adaptation phase is employed. Thereafter, the LTTs can be segmented and successively converted into definitive restorations in a stepwise manner, thereby reducing the overall treatment intensity. Owing to the strategic selection of LTT materials (such as resin nanoceramics or polymer‐infiltrated ceramics), these temporaries may remain in place during tooth preparation and serve as core buildups. The duration of the LTT phase may be adjusted according to the patient's needs and the approved indications of the chosen material. After 6 months or more with no TMJ symptoms, the adaptation process may be considered complete, and the transition to definitive restorations can proceed. Initiating the transfer process in the posterior areas helps stabilize the situation further and should precede the placement of final anterior restorations.

#### 2.4.3 2A: Bimaxillary Polycarbonate Splint to Definitive Restorations

Polycarbonate splints can be directly transferred into definitive restorations. A key advantage of this approach lies in the ability to perform the transfer and preparation processes on a jaw‐by‐jaw basis, eliminating the need for an antagonist approach (Path 1A). In this method, the polycarbonate splint remains in one jaw—typically the mandible—whereas the maxilla is restored against the anatomically shaped polycarbonate splint.

**FIGURE 11:**
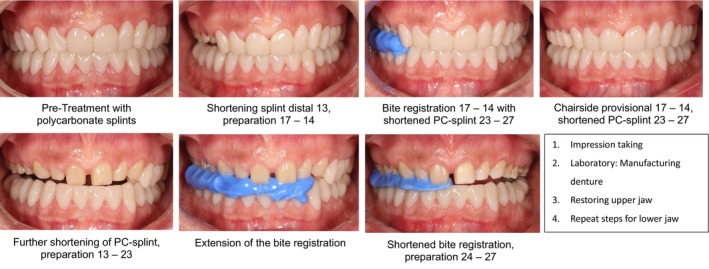
Treatment using a polycarbonate splint for upper jaw restoration (Path 2A).

Furthermore, the splint is shortened in accordance with the planned sequence of tooth preparations, providing a stable reference structure and facilitating accurate bite registration. Following each segmental preparation and the corresponding reduction of the support zone, interocclusal registration is performed. The shortened polycarbonate splint serves as a stable reference for maintaining the interocclusal distance. The prepared teeth were provisionally restored chairside at the newly established VDO. This approach enables the complete restoration of one jaw with definitive restorations prior to adapting the occlusion using the remaining splint in the opposing jaw (Figure 11). Such procedural flexibility is made possible by the anatomical design of the polycarbonate splint.

#### 2.4.4 2B: Bimaxillary Polycarbonate Splint—Long‐Term Provisionals—Permanent Restorations

The most comfortable and predictable procedure involves the placement of LTTs prior to initiating definitive restorations. Owing to the anatomical shape of the splints, a jaw‐wise approach is also possible, providing a safe and controlled method of transferring the tested splint position into definitive restorations. Nevertheless, the polycarbonate splint serves as a reference for both monitoring and reliably adjusting the occlusal relationship of the LTTs. The protocol for transferring LTTs into definitive restorations has been described previously.

## 3 Discussion

**FIGURE 12:**
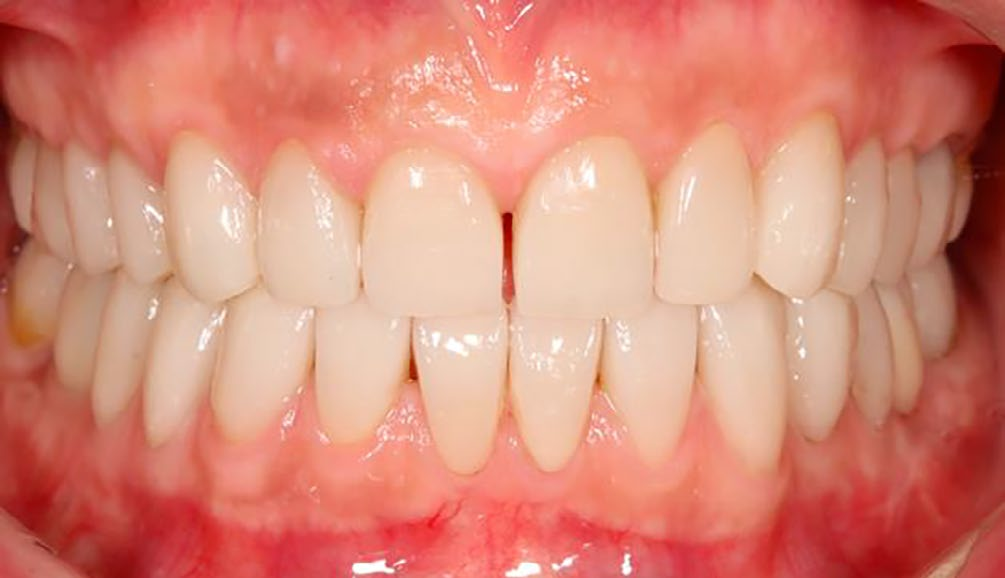
Final restoration in the same patient as shown in Figures 7 and 9 (material: e.max Press, Ivoclar).

Various methods are available for the treatment and rehabilitation of patients with a loss of VDO, each offering specific advantages and disadvantages. In summary, the management of VDO loss and compromised physiological occlusion requires extensive prosthodontic treatment. A critical aspect of this therapy is establishing a new jaw relationship, maintaining it throughout the treatment phases, and precisely and securely transferring it into definitive restorations (Figure 12).

**FIGURE 13:**
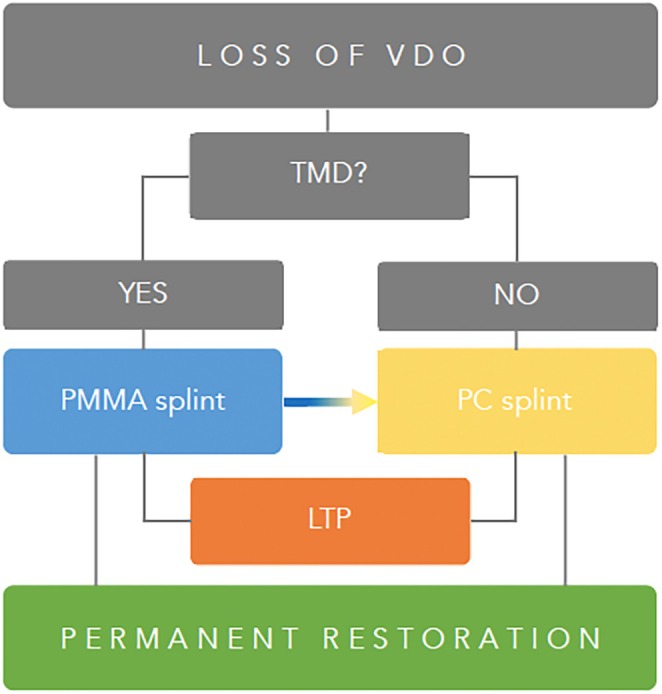
Overview of different treatment pathways from splint to final restoration.

Another key point is the initial TMJ screening to diagnose functional disorders of the TMJ and masticatory musculature, as these findings significantly influence the subsequent steps required in therapy. Dentists must determine whether the initial treatment phase should focus on testing a newly established restorative concept within a revised jaw relationship and VDO, or whether the initial step is to treat TMD. TMDs are commonly encountered in everyday practices; however, current evidence does not indicate a direct correlation between changes in the VDO and the development of TMD [10, 23, 30]. If the primary treatment objective is TMD therapy or the establishment of a new mandibular position, a monomaxillary splint is recommended to facilitate reliable mandibular positioning. Owing to its rigid material and the capacity for subtractive and additive adjustments, functional treatments are predominantly performed using a “classic” PMMA splint (Figures 6 and 10, 13). Although the PMMA splint allows for the modification and testing of jaw relationship, its transparent, “mono‐jaw” design does not permit changes in white esthetics, which may lead to reduced patient compliance [18]. The contact zone between the opposing jaw and the PMMA splint does not align with the occlusal level planned for the definitive restoration. This makes it difficult to implement prosthetics and necessitates additional adjustments for the patient.

Polycarbonate splints are a promising alternative for patients without TMDs. They offer a reliable method for evaluating new restoration morphology, considering both functional and esthetic aspects, and effectively serve as a “test drive” for the proposed restorative design. Unlike PMMA splints, which require an antagonistic preparation approach, pretreatment with polycarbonate splints allows for a jaw‐wise preparation sequence. The polycarbonate splint enables the evaluation of esthetics, whereas the functional aspects can be evaluated during the pretreatment phase [18, 19, 20]. The polycarbonate splint enables a deeper and broader “intervention” in the undercuts, which are often minimal in cases of abrasion dentition. It also offers esthetic benefits due to its color and the even coverage of the tooth surface, resulting in a more homogeneous appearance. For enhanced esthetics, versions with color gradients are now available. In the absence of functional symptoms, splint therapy should be continued for at least 3 months [17]. Generally, a trial with splints and/or provisional restorations should precede any invasive treatment when the VDO is significantly altered.

During the temporary phase, the relatively higher abrasion rates of composites compared with ceramics can be considered beneficial, as materials like polycarbonate and LTTs allow for “grinding in” of the occlusion in contrast to ceramics [31, 32].

Non‐prep LTT offers an additional option to enhance predictability and safety during the implementation phase. These restorations enable patients to speak, eat, and chew just as they would with the final restorations. As no tooth preparation is necessary, the transfer process is less demanding and remains non‐ or minimally invasive. Fixed restorations improve adaptability to new occlusal conditions more effectively compared with removable appliances. Furthermore, increasing the VDO using fixed prostheses results in fewer symptoms compared with removable temporaries or splints. Contributing factors may include improved and continuous wearing comfort, morphology that closely resembles the final restoration, and higher esthetic and patient acceptance of fixed appliances [8].

Wearing time should be determined individually according to patient‐specific factors, although it may be limited by the material's regulatory approval.

In summary, restoring VDO and physiological function involves significant treatment effort and requires a personalized approach with carefully selected procedures. Generally, the methods and techniques outlined in this study can be flexibly combined to meet the specific clinical needs of each case. The treatment plans described allow for better planning of treatment duration based on the patient's resilience and enhance predictability through a stepwise approach. Within the framework of a patient‐centered, personalized treatment approach, each case must be planned with precision and tailored individually based on detailed clinical findings and analyses.

## Conflicts of Interest

The authors declare no conflicts of interest.

## Data Availability

The data that support the findings of this study are available from the corresponding author upon reasonable request.
